# Cow’s milk allergy skin tests: fresh milk, commercial extracts, or both?

**DOI:** 10.1186/s13223-023-00763-w

**Published:** 2023-01-18

**Authors:** Idit Lachover-Roth, Nadav Giorno, Tzipi Hornik-Lurie, Anat Cohen-Engler, Yossi Rosman, Keren Meir-Shafrir, Ronit Confino-Cohen

**Affiliations:** 1 Allergy and Clinical Immunology Unit, Meir Medical Center, 44261 Kfar Saba, Israel; 2 Sackler School of Medicine, Tel Aviv University, Tel Aviv, Israel; 3 Meir Medical Center, Kfar Saba, Israel

**Keywords:** Food allergy, Skin prick test (SPT), Commercial extracts, Fresh food, Cow’s milk allergy

## Abstract

**Background:**

The diagnosis of food allergy is based on a history of immediate allergic reaction following food ingestion, and skin prick test (SPT) demonstrating sensitization with commercial extracts (CE) or fresh food (FF). For most food allergens, the SPT with FF is considered more accurate and predictive. Regarding cow’s milk, the results are inconclusive. This retrospective study aimed to evaluate the accuracy of SPT with fresh milk compared to CE (cow’s milk and casein) for evaluation of cow’s milk allergy (CMA).

**Methods:**

This study summarized the medical records of children, diagnosed with CMA. The data include demographics, skin tests and oral food challenge results, as well as atopic comorbidities.

**Results:**

Records of 698 patients with the diagnosis of CMA were reviewed, 388 fulfilled the inclusion criteria. Overall, 134 patients (34.54%) had an additional atopic disease. The SPT wheal size with fresh milk was significantly larger than with CE (cow's milk and casein) at first evaluation or before oral food challenge (OFC). Combination of SPT results (CE and FF) gave the maximal odds ratio for reaction during OFC and SPT with fresh milk alone gave the minimal OR (34.18 and 4.74, respectively).

**Conclusions:**

SPT with CE for CMA evaluation is more reliable than SPT performed with fresh milk. In patients suspected of having IgE-mediated CMA, before deciding on performing OFC, it is advised to perform SPT with at least two different extracts, and always include casein. Fresh milk can serve as a backup if commercial extracts are not available. In cases that the SPT with fresh milk is 3 mm or less, there is 93.3% chance that the OFC will pass without reaction.

*Trial registration* This study protocol was reviewed and approved by the Ethics Committee of Meir Medical Center, IRB Number 0083-18 MMC.

**Supplementary Information:**

The online version contains supplementary material available at 10.1186/s13223-023-00763-w.

## Background

Food allergies are common and present an increasing health problem, affecting up to 10% of young children [1]. The prevalence of cow’s milk allergy (CMA) is estimated to be 0.5–0.74% in the first year of life [2, 3].

The diagnosis of food allergy is based on a history of immediate allergic reaction following ingestion of the food and a skin prick test (SPT) showing sensitization and/or specific IgE. However, the gold standard for diagnosis or exclusion of food allergy is an oral food challenge (OFC) [4]. OFC is also performed when the history is inconclusive or when the SPT is equivocal.

A SPT is performed using either a commercial extract (CE) or fresh food (FF). It is considered positive when the wheal diameter is ≥ 3 mm [5]. For most food allergens, SPT with FF will cause a larger wheal diameter than CE. The age at which the SPT is performed also affects the results, with smaller wheal diameters in younger children [4, 6].

Many studies tried to define the wheal size that can predict an allergic reaction during OFC [4–8]. It is believed that SPT with FF is more predictive than that performed with CE [6, 11, 12]. Rance et al. found that for different foods, the correlation between SPT wheal size and the OFC results was higher when SPT was done with FF compared to CE [13]. Regarding cow's milk (CM), the specificity of FM SPT was 100% compared to 50% with CE. CE was more sensitive than FM (73% vs. 66%) [13]. However, specifically regarding CM, FF was not superior to CE in predicting allergic reaction during OFC, as 9 children with CE SPT wheal size > 3 mm had an allergic reaction during OFC compared to the same number of children who had wheal size > 3 mm with FF SPT. When SPT < 3 mm also the same number of children with CE SPT < 3 mm failed the OFC as children with FF SPT < 3 mm [13]. Calvani et al. defined, in patients with CMA, a cutoff for SPT as 7 mm for casein, 20 mm for cow’s milk CE and 10 mm for SPT with fresh milk (FM) [9, 10]. These cutoffs produced a specificity of 100% but the sensitivity was zero.[9] Most studies were small series and the recommendations regarding whether SPT to evaluate CMA should be performed with CE and/or FF were inconclusive.

The current study aimed to evaluate the accuracy of SPT with FM compared to CE for evaluating CMA.

## Methods

This retrospective study included children and teenagers up to age 18 years, with past or current diagnosis of CMA, who were treated at the Allergy Unit from 2010 to 2018.

Data for the entire cohort were retrieved from the Health Maintenance Organization electronic medical record system. Data collected included demographic parameters, age and symptoms during the index reaction, size of SPTs at all evaluations. Eosinophil count proximity to the index reaction, OFC results, current allergic status and atopic comorbidities: asthma, atopic dermatitis, allergy to other foods, and family history of atopic diseases were recorded. The allergic rhinitis symptom as an atopic comorbidity was excluded in the analyses due to the young age of the patients and therefore low and biased prevalence of this comorbidity.

Children and teenagers up to age 18 years, with a history of suspected event as immediate allergic reaction to CM products and SPT confirming the diagnosis were included in the study.

Patients with anamnestic details implying late reaction or without at least one positive SPT were excluded.

SPT were performed by trained staff on the volar aspect of the forearm, with commercial CM extracts (1:10 W/V, ALK-Abello Pharm. Inc), casein (1:100 W/V, ALK-Abello Pharm. Inc), and fresh CM (3% fat). Positive (histamine 1 mg/ml) and negative (0.9% normal saline) controls were also performed. SPT were defined as positive, for all patients in all ages, when the wheal diameter was at least 3 mm larger than the wheal size of the negative control after 15 min. The SPT results that were taking into analyses were the first and last SPT that were done, even if the subject has done more than two SPT's. For children who underwent OFC, the last SPT taken in account was the SPT done before the OFC. For children who were defined as allergic without OFC, the last SPT was used as defining them as allergic for all the analyses.

The decision to perform an OFC was made by an allergy specialist according to clinical parameters and SPT results. OFC was an open challenge with CM formula for infants younger than 12 months and FM for toddlers and children above 12 months.

### Challenge protocol

The first dose of 0.5 ml was doubled every 30 min until a final dose of 80/160 ml, containing 2.72/5.44 g total milk protein, respectively was obtained. Toddlers younger than 2 years of age received final dose of 80 ml. Between 2 and 5 years the final dose depends on the child cooperation. OFC was stopped and considered as a failure when one of the following symptoms appeared: urticarial rash, cough, wheezing, or vomiting.

The study cohort was divided into three groups: A. Allergic by OFC or SPT: A.1 Allergic by OFC-patients who referred to OFC by their allergologist due to lack of allergic reactions within few years or improvement in the SPT results and had an allergic reaction during OFC. A.2. Allergic by SPT-patients with high suspicion of existing CMA according to the decision of the patients' allergologist, based on allergic reaction following accidental exposure lately and/or large wheal diameter in the SPT. B. Not allergic according to OFC.

### Statistics

Data were analyzed using SPSS/PC, version 25.0. Descriptive statistics were used to characterize the study participants. Chi-square and one-way ANOVA tests were employed to examine differences in demographic and clinical characteristics between the three groups (A1, A2 and B). OR and 95%CI were calculated.

A hierarchical, binary logistic regression was performed to determine the relationship between demographic variables (sex and age); other comorbidities (asthma, atopic dermatitis, other food allergies), family atopic background, symptoms during the first reaction at the categorical level (yes/ no), and continuous parameters (age at reaction, SPT result, eosinophil count). Two-by-two tables were used to calculate sensitivity, specificity, positive predictive values (PPV), negative predictive values (NPV) and Odds Ratio (OR) of the last SPT values.

To define the sensitivity and specificity of the SPT ratio of milk extract, casein and FM to detect the CMA, a receiver-operating characteristic (ROC) curve was plotted. Cut-off levels were optimized (sensitivity + specificity). The accuracy was measured by area under the curve (AUC) analysis. The OR was calculated by binary logistic regression model. Two-sided tests of significance p < 0.05 were used in all analyses.

The study was approved by the local Ethics Committee.

## Results

**Fig. 1:**
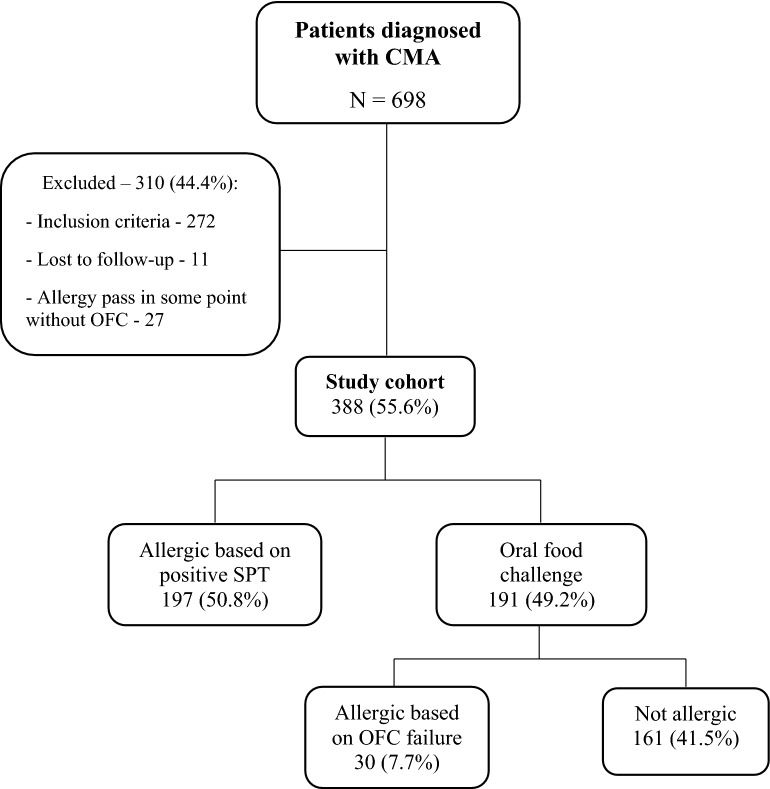
Patient flow diagram. *CMA* cow's milk allergy; *OFC* oral food challenge; *SPT* skin prick test

From 2010 through 2018, 698 patients with a diagnosis of "CMA", were evaluated. Among them, 272 (39.0%) were excluded based on the exclusion criteria, and another 38 were lost to follow-up (shown in Fig. 1). Of the 388 patients in the study cohort, 215 (55.4%) were male.

The study cohort of 388 patients included the following subgroups: Group A (allergic) 227(58.5%), of whom 30(7.7%) were proven by OFC (group A1), and 197(50.8%) by SPT (group A2). Group B (non-allergic) included 161 subjects (41.5%) who did not react during the OFC.

There were no significant differences between the three groups regarding age at the first allergic reaction (shown in Table 1). Additional demographic parameters are presented in Table 1.

**Table 1:** Comparison of demographic and clinical parameters between the 3 study groups

	Total (n = 388)	Allergic—total (Group A) (N = 227)		(B) Not allergic by OFC (Group B) (n = 161)
		Allergic by OFC (A.1) (n = 30)	Allergic by SPT (A.2) (n = 197)	
Male sex, n (%)	215 (55.4)	139^a^ (61)		76^b^ (47.5)
		20^a,b^ (64.5)	119^a^ (60.4)	
Age at first exposure, (months) mean ± SD (95% CI)	3.32 ± 3.29 (2.93–3.7)	3.44 ± 3.38^a^ (2.93–3.95)		3.12 ± 3.16^a^ (2.53–3.72)
		3.69 ± 2.93^a^ (2.45–4.93)	3.4 ± 3.45^a^ (2.84–3.96)	
Age at first reaction (months), mean ± SD (95% CI)	5.04 ± 2.89 (4.74–5.35)	5.03 ± 3.06^a^ (4.61–5.45)		5.07 ± 2.64^a^ (4.63–5.5)
		4.47 ± 2.51^a^ (3.51–5.42)	5.1 ± 3.14^a^ (4.64–5.56)	
Age at last SPT (years), mean ± SD (95% CI)	4.43 ± 3.06 (4.09–4.76)	5.27 ± 3.39^a^ (4.76–5.78)		3.46 ± 2.29^b,§^ (3.09–3.83)
		4.82 ± 3.92^a,§^ (3.3–6.34)	5.36 ± 3.28^a^ (4.82–5.9)	
Asthma, n (%)	82 (21.13)	68^a^ (30)		14^b,§^ (8.75)
		7^a,§^ (22.58)	61^a^ (31.12)	
Atopic dermatitis, n (%)	68 (17.52)	41^a^ (18.1)		27^a^ (16.9)
		6^a^ (19.35)	35^a^ (17.7)	
Other food allergy, n (%)	78 (20.1)	55^a^ (24.2)		23^b^ (14.4)
		5^a,b^ (16.1)	50^a^ (25.5)	
Family atopic background, n (%)	185 (52.4)	117^a^ (54.4)		68^a^ (49.3)
		17^a^ (58.6)	100^a^ (53.8)	
Symptoms during the first reaction, n (%)				
Rash	335 (86.3)	203^a^ (91)		132^a^ (84.6)
		27^a^ (87.1)	176^a^ (91.7)	
Breathing difficulties	55 (14.5)	48^a^ (21.5)		7^b^ (4.5)
		4^a,b^ (12.9)	44^a^ (22.9)	
Vomiting	212 (55.9)	132^a^ (59.2)		80^a^ (55.9)
		18^a^ (58.1)	114^a^ (59.4)	
Eosinophils at diagnosis mean ± SD (95% CI)	487.74 ± 520.66 (435.57–539.91)	546.23 ± 595.12^a^ (468.57–623.89)		402.8 ± 373.72^b^ (343.89–461.72)
		431.29 ± 335.1^a,b^ (308.37–554.21)	564.31 ± 624.96^a^ (476.5–652.13)	

Superscripts indicate significant differences p < 0.05 (for three categories, the superscripts indicate significant differences in post hoc tests p < 0.05, for Chi-square tests, a z-test with Bonferroni correction for comparison of column proportion, and for One-way ANOVA Tukey HSD to examine average differences between all pairs). ^§^p < 0.1

*SD* standard deviation, *OFC* oral food challenge, *SPT* skin prick test

### Atopic comorbidities

Overall, 134 patients (34.5%) had at least one additional atopic disease; 82(21.1%) had asthma and 68 (17.5%) had atopic dermatitis. Food allergies other than milk were found in 78 patients (20.1%). The prevalence of asthma was similar between group A1 and A2 (p = NS). The incidence in groups A1 and A2 compared to group B was statistically significant (22.58% for group A1 and 31.12% for group A2 vs 8.75% for group B; p < 0.05).

The incidence of other food allergy was significantly more common in group A2 when compared to group B (25.5% vs 14.4%, respectively p < 0.05) (shown in Table 1).

### Symptoms during the first reaction

Urticarial rash was the most common symptom affecting 335 (86.3%) patients, 55 (14.5%) had breathing difficulties and 212 (55.9%) had vomiting. Breathing difficulties were more prevalent in group A2 as compared to group B (22.9% vs 4.5%, p < 0.001; shown in Table 1). No other symptoms were reported.

### Skin tests

All subjects (n = 388) had done SPT with milk extract and casein simultaneously. Of them, 322 (83%) subjects had done also SPT with FM. From the entire cohort, 66 (17%) were examined only once and have results for the first SPT alone. From those who had last SPT’s, 274 (85%) had done also SPT with FM (shown in Additional file 1: Table S1).

#### Skin test with FM

**Fig. 2:**
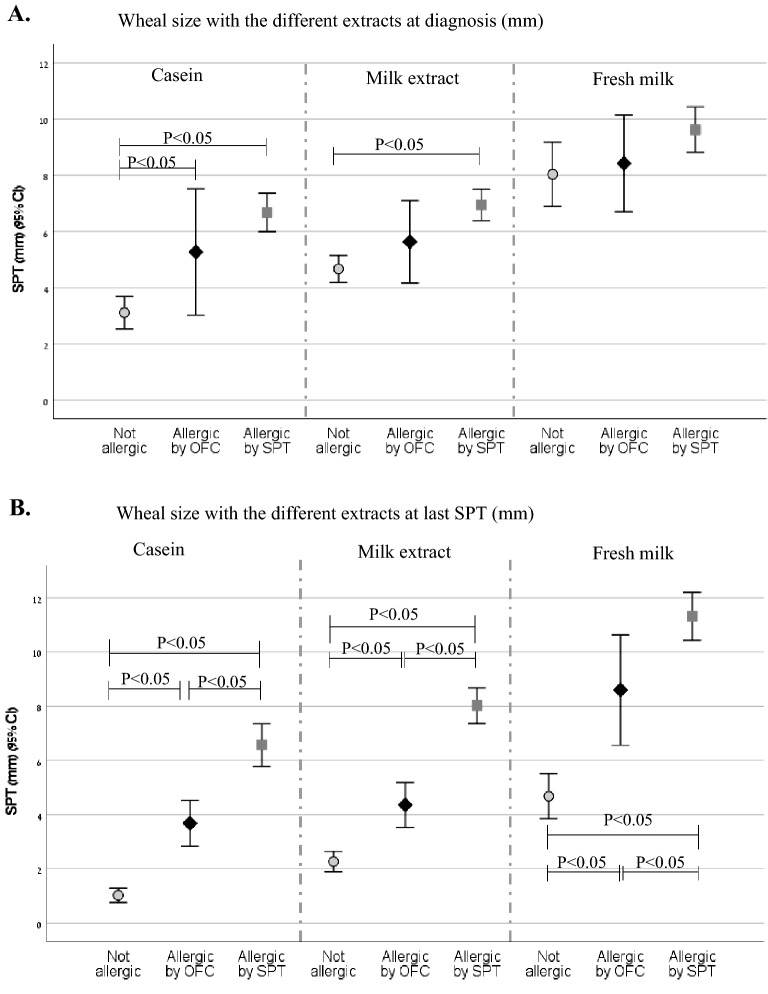
Skin prick tests wheal size (mm) in the different study groups. **A** Mean skin prick test results at diagnosis. **B** Mean last skin prick test results. Only significant differences are shown

The wheal size with FM was significantly larger than with milk extract and casein at the first SPT recorded and in the last SPT recorded in the total cohort and for each group separately: at the first SPT—5.1 mm, CI 95%(4.62–5.6) vs. 5.91 mm, CI 95%(5.53–6.3) vs. 8.88 mm, CI 95%(8.24–9.52), p < 0.05, for casein, milk extract and FM respectively. At the last SPT—3.74 mm, CI 95%(3.26–4.21) vs. 5.02 mm, CI95%(4.56–5.48) vs. 8.21 mm, CI 95%(7.52–8.9), for casein, milk extract and FM respectively; p < 0.05 (shown in Fig. 2).

#### First SPT

The time elapsed between the first recorded reaction and the first SPT was 11.51 ± 0.72 months.

The mean SPT results of the first tests are shown in Fig. 2A. Significant differences between the wheal size in the different groups were found only in SPT with casein (5.27 mm, CI 95%(3.02–7.52), 6.68 mm, CI 95%(5.99–7.37), 3.12 mm, CI 95%(2.53–3.7), p < 0.05 for groups A1, A2, and B, respectively). Significant differences in the wheal size of SPT with milk extract were found only between group B and group A2 (4.67 mm, CI 95%(4.19–5.15) vs. 6.95 mm, CI 95%(6.39–7.51), respectively). There were no significant differences between the wheal size in the first SPT with FM among the groups (8.43 mm, CI 95%(6.71–10.15), 9.63 mm, CI 95%(8.82–10.44), 8.04 mm, CI 95%(6.9–9.18) for groups A1, A2, and B, respectively).

Only two patients in the not allergic group (1.25%) had first casein SPT ≥ 14 mm.

A binary adjusted logistic regression models have shown significant association between the wheal size of the first SPT with casein and the OFC results (OR = 1.148, CI 95%(1.046–1.259), p < 0.05; shown in Additional file 1: Table S2A). These results are in line with the unadjusted results.

#### Last SPT

The time elapsed between the first recorded SPT and the last was 2.48 ± 0.13 years.

The mean SPT results of the last tests recorded are shown in Fig. 2B. For patients who underwent OFC, the last SPT results are those prior to the OFC. Significant differences between the wheal sizes in the different groups were found in SPT with all the extracts, including FM.

Logistic regression models after adjustment showed that the association between the wheal size of the last SPT with each extract and the OFC results was statistically significant (shown in Additional file 1: Table S2B). However, the OR for casein was significantly higher than the OR of milk extract and FM (1.907, 1.354 and 1.151, respectively).

#### Ratio between the first SPT and the last SPT

**Fig. 3:**
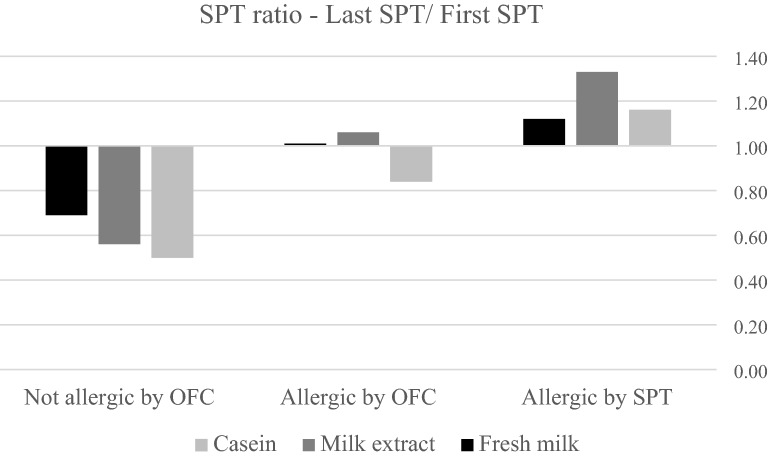
The ratio between the last skin prick test and the first skin prick test recorded according to the study groups. *OFC* oral food challenge; *SPT* skin prick test. The valid number of SPT for calculations—Not allergic (casein—145, milk extract—147, fresh milk—118), allergic by OFC (casein—27, milk extract—28, fresh milk—25), and allergic by SPT (casein—140, milk extract—143, fresh milk—129)

The ratio $\frac{Last \,SPT \,wheal \,size\,(mm)}{First \,SPT\, wheal \,size\,(mm)}$ was < 1 for casein, milk extract, and FM in group B, and casein in group A1. The ratio was > 1 for all extracts in group A2 and milk extract and FM in group A1 (shown in Fig. 3). The ratio was significantly lower in group B compared to the two other groups for all three types of SPT extracts (p < 0.05), except for FM where the difference was significant between groups B and A2, and not between groups B and A1. There were no significant differences between the two allergic groups. Logistic regression models after adjustment showed that each extract ratio was statistically significant. The maximal OR was 2.922 (CI 95% (1.317–6.481), p < 0.01) for casein SPT ratio, but without significant differences for SPT ratio with milk extract and FM (shown in Additional file 1: Table S2C).

#### Cutoff point for the different extracts

Standardization of wheal size to age led to the equations:

$${\text{Milk extract wheal size}}\left( {{\text{mm}}} \right) \, = {1}.{175} + \left( {0.{316}*{\text{age}}} \right),$$

$${\text{FM wheal size}}\left( {{\text{mm}}} \right) = {2}.{8}0{2} + \left( {0.{525}*{\text{age}}} \right).$$

For casein, the correlation between age and wheal size was not significant, and wheal size < 3 mm was defined as negative for all ages.

From this point, negative and positive SPT results were defined accordingly.

The NPV of all extracts separately or in combination ranged 92.76–94.2%, without significant differences. The PPV was maximal with the combination of all extracts with 72.73%, the sensitivity was maximal with FM 84%, and the specificity was maximal with the combination of all three extracts with a rate of 95.92% (range 55.08–95.92%). The OR was maximal for the combination of all three extracts and minimal with FM (34.18, 6.44 respectively) (shown in Table 2). NPV, PPV, sensitivity, and specificity were calculated only for groups A1 and B.

**Table 2:** Predictive values of the different extracts

	Casein	Milk extract	Fresh milk	All extract	Combination of casein and milk extract	Combination of casein and fresh milk
NPV (%)	93.2	93.97	93.33	92.76	92.76	93.24
PPV (%)	58.06	33.87	25.3	72.73	65.38	68
Sensitivity (%)	64.29	75	84	59.26	60.71	62.96
Specificity (%)	91.33	72.67	47.46	95.92	94	94.52
OR	18.97	7.97	4.74	34.18	24.21	29.33

Predictive values of the different extracts in the last skin prick test recorded with a cutoff of SPT ≤ 3 mm as negative result and SPT > 3 mm as positive result for milk extract and casein, and fresh milk. All extract = at least one of the SPT results was negative

The predictive values, sensitivity, specificity, and OR were calculated only for patients who underwent oral food challenge (Groups A1 and B)

**Fig. 4:**
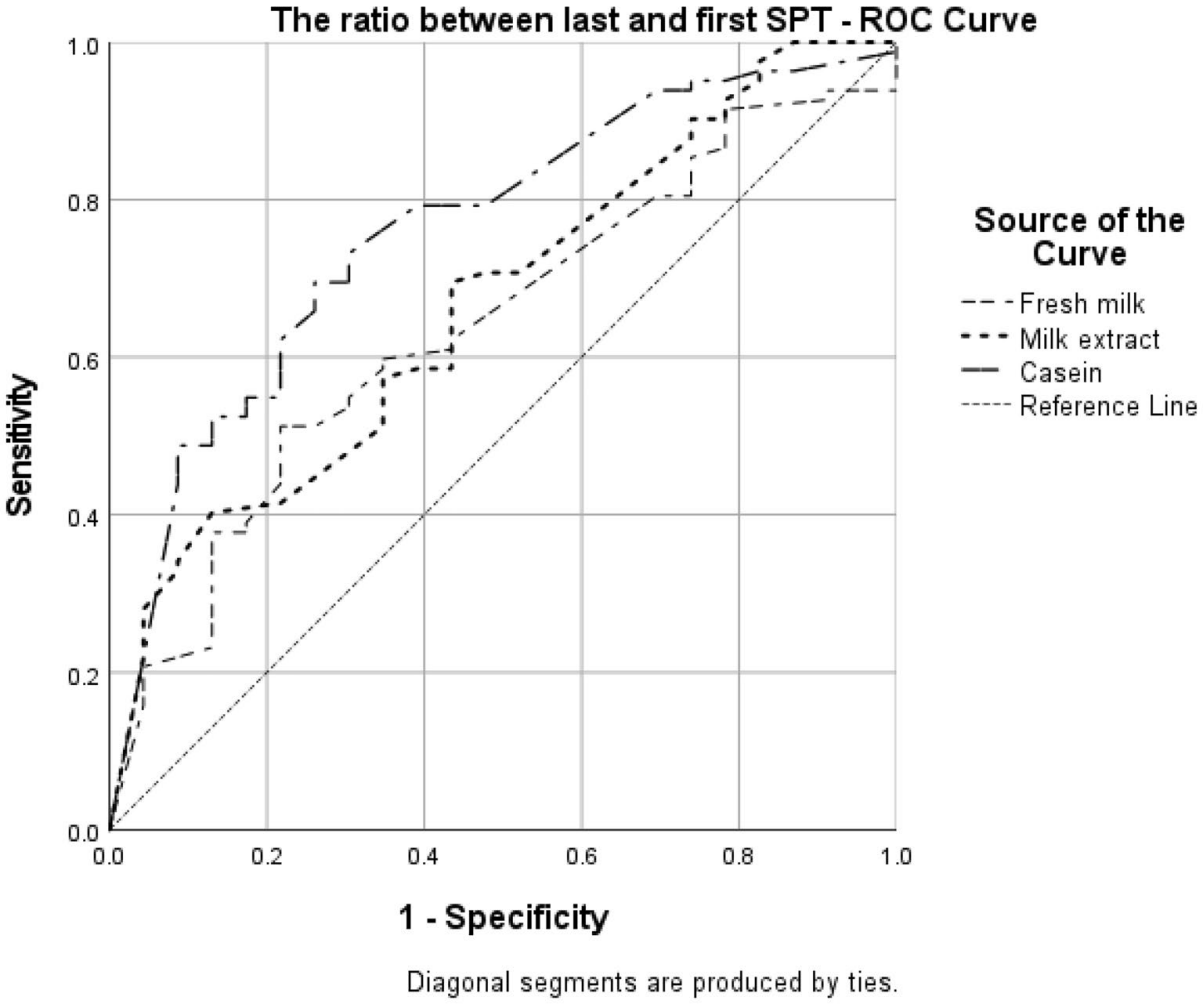
ROC curve for the ratio between the last and the first SPT with the different extracts. ROC curve created for groups A1 and B (Allergic by oral food challenge and Not allergic by oral food challenge)

The AUC for SPT ratio of milk extract and FM were under the accepted value for discrimination (0.67 and 0.68 respectively). The AUC for SPT ratio of casein was 0.73. ROC curve analyses for the ratio found that the optimal sensitivity and specificity for the SPT ratio with casein was 0.68 (OR = 5.36, p < 0.001; shown in Fig. 4). There was no optimal value for milk extract and FM. The ROC curve was created for groups A1 and B, excluding group A2 that was defined as allergic without OFC.

## Discussion

Food allergy is an increasing health problem, especially in children. The accepted follow-up for allergic children includes periodical SPT with the food allergen. The most available extract for SPT is FF. However, its reliability is questionable and differs between foods. In peanuts, egg, tree nuts, and fruits, the FF SPT gave more reliable results with sensitivity and specificity at least as good as the CE [8, 9, 13]. The current study aims to assess the advantages and drawback of each SPT method, and give the physician opportunity to examine their current practice. Our study demonstrated that in the case of CM, the commercial milk extract and casein are more accurate than FM is, and casein alone is superior to the others. A combination of the results from all three extracts, or at least a combination of two extracts, is the most accurate way to decide who has the best chance to pass OFC without an allergic reaction, and with the highest sensitivity and specificity. FM can serve as a backup if CE are not available. If the SPT with FM is ≥ 4 mm, the OR is 4.74 to develop reactions during OFC; while if the SPT is ≤ 3 mm, there is a 93.33% chance that the OFC will pass without reaction. The low specificity is the drawback of SPT with FM, which is the main reason it is not recommended as the sole extract for SPT.

The cutoff for negative results with CE yields maximal sensitivity and specificity when the wheal is ≤ 3 mm. Previous studies recommended higher cutoff points, with higher specificity, but with higher rate of reactions during OFC with minimal gain [5–7]. Therefore, we recommend using the safer cutoff. It is important to emphasize that those cutoffs, based in the results of our study, are relevant only for subjects who had immediate symptoms following consumption of dairy product and are suspected as allergic to cow's milk or as a follow-up after children who had diagnosis of CMA to decide whether it is safe to challenge them. Regarding children with mild and uncertain symptoms, more studies need to be done to assess the reliability of those cutoffs.

The ratio between the last SPT performed to the first SPT can also point toward the chance to pass the OFC. Logistic regression models have shown a maximal OR with casein ratio. The ROC curve analyses showed that the cutoff for the SPT ratio with casein gives maximal sensitivity and specificity when the ratio is > 0.68, with OR = 5.36 for an allergic reaction during the OFC. Nonetheless, the added value beyond looking at the last SPT is small. In borderline cases, it can support the decision to perform or to postpone OFC.

One of the most common questions that parents of allergic patients ask is the chance that the CMA will resolve. Looking at the SPT result with casein from the first allergic evaluation can give a clue regarding the chance that the CMA will wane over time. The higher the initial SPT with casein, the lower the chance for the patient to outgrow the CMA. When the first casein SPT is ≥ 14 mm, the likelihood of developing tolerance in the following three years is exceedingly small. A longer follow-up is needed to assess if casein SPT ≥ 14 mm is a negative prognostic factor for overall recovery rate. As opposed to the study by Uncuoglu et al.  [7], in our study, the first SPT with FM did not predict the chance for developing tolerance in the future. However, further studies are needed to create a risk table.

Age affects the wheal size mainly with FM as shown in the equations we calculated. The influence of age when we applied those equations is minimal and not significant, then for clinical purposes we recommend using fixed cutoffs for all ages.

Some clinical parameters were significantly more prevalent in patients who failed the OFC, but multivariate analyses did not find reliable clinical score models that could calculate all variables to define which patients have a high chance to pass an OFC without reaction. The probable reason is that the weight of the SPT results of milk extract and casein is highly significant and overshadows the other parameters.

To avoid bias, most of the statistical analyses were performed on the data of patients who underwent OFC. However, the similarity in the clinical parameters between patients allergic by SPT (group A2) to those defined as allergic by OFC (group A1), and the difference from the not allergic group (group B), was enough to justify the “CMP allergy” label of group A2. According to our results, it is reasonable to assume those patients would have fail in OFC, but as we did not challenge them, we cannot be 100% sure.

This study had limitations inherent to its retrospective nature. The definition of "breathing difficulties" was based on the reports written in the medical record and it is difficult to define more precisely. A second limitation is related to the inclusion criteria. We deliberately excluded from the study children without at least one positive SPT results and diagnosis of CMA. This exclusion caused an intention bias toward allergic children and therefore we cannot assess the NPV of SPTs with CE or FM in the general pediatric population addressing for allergy evaluation with suspected allergic reaction to cow's milk. Focusing on this specific population, especially those who underwent OFC, made our results more accurate in assessing the value of CE and FM. If the SPT results can predict accurately the recovery from CMA, we can assume that it can assess accurately children with low suspicious for CMA, but further studies are needed. Moreover, there was a bias in the patients selected for OFC, probably in favor of those who had a good chance to pass the OFC without an allergic reaction, and as OFC is the "gold standard" it is possible that we labeled non-allergic patients as allergic (group A2). Nevertheless, there were significant differences between the not allergic patients and those allergic by OFC, with no significant differences between the patients allergic by OFC or SPT.

In conclusion, SPT serve as a decision support tool to decide which patient has a good chance to pass OFC without allergic reaction, but it cannot replace OFC. In patients suspected of having IgE-mediated CMA, before deciding on performing OFC, it is advised to perform SPT with at least two different extracts, and always include casein. When the only material available for SPT is FM, it can give a good sense of who has a good chance to pass OFC without allergic reaction. Further prospective studies are required to strengthen these findings.

Who is the best candidate for OFC?

1. SPT wheal size ≤ 3 mm with casein and/or milk extracts and/or fresh milk.
2. Casein wheal size ratio $\frac{Last \,SPT\, wheal\, size (mm)}{First \,SPT\, wheal \,size (mm)}$<0.68.

## Supplementary Information

**Additional file 1: Table S1.** Number of subjects done SPT first and last. **Table S2.** Logistic regression models for the different skin prick test results.

## Data Availability

All data generated or analyzed during this study are included in this article. The dataset used and/or analyzed during the current study are available from the corresponding author on reasonable request.

## Abbreviations

CE: Commercial extracts
CM: Cow’s milk
CMA: Cow’s milk allergy
FF: Fresh food
FM: Fresh milk
OFC: Oral food challenge
SPT: Skin prick test
