# Supplementary material for: Cow’s milk allergy skin tests: fresh milk, commercial extracts, or both?
Source: Allergy Asthma Clin Immunol. 2023 Jan 18;19:6. doi: 10.1186/s13223-023-00763-w (PMC9847062; doi:10.1186/s13223-023-00763-w)
Supplement: Supplementary file 1 — Additional file 1: Table S1. Number of subjects done SPT first and last. Table S2. Logistic regression models for the different skin prick test results. [file 13223_2023_763_MOESM1_ESM.docx]

Table S1: Number of subjects done SPT first and last:

|  | | **Group A1**  (n=30) | **Group A2**  (n=197) | **Group B**  (n=161) |
| --- | --- | --- | --- | --- |
| **First SPT** | Milk extract | 30 | 197 | 161 |
|  | Casein | 30 | 197 | 161 |
|  | Fresh milk | 29 | 169 | 124 |
| **Last SPT** | Milk extract | 27 | 144 | 151 |
|  | Casein | 27 | 144 | 151 |
|  | Fresh milk | 25 | 131 | 118 |

Table S2: Logistic regression models for the different skin prick test results

**A.** Absolute wheal size of the different first recorded SPT

| Model | Independent variables | B | Odds Ratio | 95% CI for OR | | p | Nagelkerke pseudo r^2^ (adjusted^*^) | Nagelkerke pseudo r^2^ (overall) | χ^2^ (7) | p |
| --- | --- | --- | --- | --- | --- | --- | --- | --- | --- | --- |
|  |  |  |  | Lower | Upper |  |  |  |  |  |
| 1 | Fresh milk | 0.054 | 1.056 | 0.977 | 1.141 | 0.172 | 0.132 | 0.15 | 14.561 | 0.042 |
| 2 | Milk extract | 0.108 | 1.114 | 0.983 | 1.263 | 0.092 | 0.088 | 0.112 | 12.772 | 0.078 |
| 3 | Casein | 0.138 | 1.148 | 1.046 | 1.259 | **0.003** | 0.088 | 0.162 | 18.636 | 0.009 |

**B.** Absolute wheal size of the different last recorded SPT

| Model | Independent variables | B | Odds Ratio | 95% CI for OR | | p | Nagelkerke pseudo r^2^  (adjusted) | Nagelkerke pseudo r^2^ (overall) | χ^2^ (7) | p |
| --- | --- | --- | --- | --- | --- | --- | --- | --- | --- | --- |
|  |  |  |  | Lower | Upper |  |  |  |  |  |
| 1 | Fresh milk | 0.141 | 1.151 | 1.043 | 1.272 | **0.005** | 0.125 | 0.211 | 19.288 | 0.007 |
| 2 | Milk extract | 0.303 | 1.354 | 1.129 | 1.624 | **0.001** | 0.11 | 0.212 | 22.984 | 0.002 |
| 3 | Casein | 0.646 | 1.907 | 1.497 | 2.429 | **<0.001** | 0.11 | 0.408 | 47.201 | 0.000 |

**C.** The ratio between the last SPT results and the first SPT recorded.

| Model | Independent variables | B | Odds Ratio | 95% CI for OR | | p | Nagelkerke pseudo r^2^  (adjusted) | Nagelkerke pseudo r^2^ (overall) | χ^2^ (7) | p |
| --- | --- | --- | --- | --- | --- | --- | --- | --- | --- | --- |
|  |  |  |  | Lower | Upper |  |  |  |  |  |
| 1 | Fresh milk ratio | 0.779 | 2.178 | 0.939 | 5.053 | 0.07 | 0.125 | 0.16 | 14.45 | 0.044 |
| 2 | Milk extract ratio | 0.904 | 2.468 | 1.337 | 4.558 | **0.004** | 0.109 | 0.2 | 21.275 | 0.003 |
| 3 | Casein ratio | 1.072 | 2.922 | 1.317 | 6.481 | **0.008** | 0.119 | 0.186 | 19.355 | 0.007 |

All models adjusted for gender, age at performing the SPT, asthma, food allergy other than milk, a symptom of breathing difficulties at the first allergic reaction, and eosinophil count.

^*^Nagelkerke pseudo r^2^ (Adjusted) – only for the adjusted variables.
